# 

**Published:** 1879-01

**Authors:** 


### Books and Pamphlets Received.

A Manual of Physical Diagnosis. By Francis Delafield, M. D., and Chas. F. Stillman, M. D. Wm. Wood & Co.

Lectures on Localization in Diseases of the Brain. Delivered at the Faculté De Médicine, Paris, 1875. By J. M. Charcot. Translated by Edward P. Fowler, M. D., New York. Wm. Wood & Co., New York H. H. Otis, Buffalo.

The Principles and Practice of Surgery. By John Ashurst, Jr. M. D. Second Edition. Henry C. Lea, Philadelphia. T. H. Butler, Buffalo.

Lectures on Bright's Disease of the Kidneys. Delivered at the School of Medicine of Paris, by J. M Charcot, translated by Henry D. Millard M. D., A. M. Wm. Wood & Co., New York. H. H. Otis, Buffalo.

Transactions of the Thirty-Third Annual Meeting of the Ohio State Medical Society, held at Columbus, May 14th, 15th and 16th, 1878.

The Testimony of Medical Experts. Annual Address of W. H. Philips, M. D., Kenton O., retiring President of O. State Medical Society.

An Abstract of the Laws of the State of New York, in regard to the Commitment of Insane to Asylums, their Detention and Discharge, and Comparison of the same, with the Statutory Provisions of England. By John P. Gray, M. D., L.L D., Medical Superintendent of N. Y. State Lunatic Asylum, Utica.

Report of the Quebec Lunatic Asylum for 1876, 1877, also 1877, 1878.

Ninety-Sixth Annual Catalogue of the Medical School (Boston) of Harvard University, 1878–1879.

The Duties of the Medical Profession concerning Prostitution and its allied vices. Being the Oration before the Maine Medical Association at its Annual Meeting, 12th of June, 1878. By Frederick Henry Gerrish, M. D.

Address delivered before the American Medical Association at its Twenty-ninth Annual Session, held at Buffalo, June 4th to 7th, 1878. By T, G. Richardson, M. D., of New Orleans, President of the Association.

Transactions of the Medical Society of the State of West Virginia.

A Case of Acute Peurperal Inversion of the Uterus. A Clinical Description of a new instrument successfully employed, with remarks on the Mechanism of Restoration. By John Byrne, M. D., M. R. C. S. E.

Laceration of the Cervix Uteri. The Address in Obstetrics: Delivered before the Medical Society of the State of Pennsylvania. By Wm. Goodell, A. M., M. D.

On Gastro-Elytrotomy. By Henry J. Garrigues, M. D.

The Quarterly Journal of Inebriety—Hartford, Conn.

An Illustrated Catalogue of Microscopes and other Scientific Instruments, manufactured by R. & J. Buck, London, Eng.

Annual Report of the Pennsylvania Free Dispensary for Skin Diseases, 920 Chestnut St., Phil.

Fourth Annual Report of the Officers and Superintendent of Asylum at Walnut Hill, Hartford, Conn.; also Petition to the Legislature.

Twenty-third Annual Report of the Trustees of the State Lunatic Hospital at Northampton, Mass.

A Series of American Clinical Lectures, edited by E. C. Segiun, M. D. Vol. 3, No. 11. Two Lectures on Lister's Antiseptic Method of treating Surgical Injuries, by James L. Little, M. D.

A Series of American Clinical Lectures, edited by E. C. Seguin, M. D. Vol. 3, No. 13. The Diagnosis of Progressive Locomotor Ataxia, by E. C. Seguin, M. D.

The Scientific Lessons of the Mollie Fancher case, by Geo. M. Beard, M. D., New York.

917

### THE BUFFALO

### MEDICAL AND SURGICAL

THE BUFFALO

MEDICAL AND SURGICAL JOURNAL.

PUBLISHED MONTHLY,—Containing Original Articles, Reports of Medical Societies and Hospitals, Editorial, Reviews, Correspondence, Army News, etc., etc; including the usual variety of Medical Periodical Publications. Specimen copies sent on application to BUFFALO MEDICAL AND SURGICAL JOURNAL, J. F. MINER, M. D., 978 Main Street, BUFFALO, N. Y.

TERMS OF SUBSCRIPTION, $3 00 PER YEAR, IN ADVANCE,—All communications for publication should be sent before the fifteenth (15) of each month, or they will of necessity lie over until the next number. No change can be made in advertisements after the twenty-fifth.

Correspondence and original articles are solicited. Publication is no evidence that the views of the author are endorsed by the Journal.

